# Dynamics of telopodes (telocyte prolongations) in cell culture depends on extracellular matrix protein

**DOI:** 10.1007/s11010-014-2215-z

**Published:** 2014-09-21

**Authors:** Cristina Mariana Niculite, T. M. Regalia, Mihaela Gherghiceanu, R. Huica, Mihaela Surcel, C. Ursaciuc, M. Leabu, L. M. Popescu

**Affiliations:** 1 Department of Cellular and Molecular Biology, “Victor Babeş” National Institute of Pathology, Bucharest, Romania; 2 Department of Cellular and Molecular Medicine, “Carol Davila” University of Medicine and Pharmacy, Bucharest, Romania; 3 Laboratory of Electron Microscopy, “Victor Babeş” National Institute of Pathology, Bucharest, Romania; 4 Department of Immunology, “Victor Babeş” National Institute of Pathology, Bucharest, Romania; 5 Division of Advanced Studies, “Victor Babeş” National Institute of Pathology, 99-101 Splaiul Independentei, Sector 5, 050096 Bucharest, Romania

**Keywords:** Telocyte, Telopodes, Collagen, Fibronectin, Laminin, Time-lapse videomicroscopy, Cell layer impedance

## Abstract

Telocytes (TC) are cells with telopodes (Tp), very long prolongations (up to 100 μm) with an uneven caliber (www.telocytes.com). Factors determining the dynamics of cellular prolongations are still unknown, although previous studies showed telopode motility in TC cultures. We comparatively investigated, by time-lapse videomicroscopy, the dynamics of Tp of mouse heart TC seeded on collagen, fibronectin, and laminin. Under our experimental conditions, TC and fibroblasts (cell line L929) behaved differently in terms of adherence, spreading, and prolongation extension. Fibroblasts showed lower spreading on the matrix proteins used. The time needed for spreading was 2–4 h for TC, versus 8–10 h for fibroblasts. The values for final cell surface area after spreading were between 200 and 400 μm^2^ for fibroblasts and 800–2,000 μm^2^ for TC. TC showed a more than three times higher ability to spread on the tested matrix proteins. An extremely low capacity to extend prolongations with lengths shorter than cell bodies was noted for fibroblasts, while TC extended prolongations longer than the cell body length, with a moniliform appearance. The stronger adherence and spreading were noted for TC seeded on fibronectin, while the lowest were on laminin. Collagen determined an intermediate adherence and spreading for TC, but the highest dynamics in Tp extensions. In conclusion, TC behave differently than fibroblasts in terms of adherence, spreading, and cell prolongation extension when seeded on various matrix proteins in cell culture.

## Introduction

Telocytes (TC) are interstitial cells extending very long cytoplasmic processes named telopodes (Tp) [1], exhibiting moniliform appearance, due to podomers and podoms [2]. TC are considered cells which integrate tissue components for a coherent function by intercellular signaling following different mechanisms [3], by stromal synapses [3, 4], and by ectosomes [5]. There are several reports suggesting these cells’ commitment in tissue regeneration [6–12]. Therefore, the telocytes’ ability to extend Tp and the dynamics of these prolongations seem to be important for the cell function, including their activity in regenerative events. Dynamics of telocytes’ Tp is a challenging topic due to their ability to facilitate homo- and hetero-cellular contacts, which modulate the functions of other cells (e.g., myocytes, immune cells, stem cells, epithelial and nerve cells) in various organs [4, 13–15]. Furthermore, TC are proven to be different from fibroblasts and mesenchymal stem cells in terms of gene profile and proteomics [16–18], and even in microRNA expression [19]. Moreover, the interest for modulating the dynamics of telocytes’ Tp increases continuously due to the possibility to use cell behavior as a target in tissue regeneration [20]. Dynamics of Tp could follow mechanisms similar to those acting in cell motility, but no reported results exist so far for TC.

Cell motility is a highly dynamic event, subtly tuned, carefully regulated, accompanied by cell shape changes, and cytoskeleton reorganization, under a rigorous control and modulation by complex mechanisms involving cell adherence, spreading, and reversibility of integrins to matrix proteins interactions controlled by integrin cytoplasmic tail interactions with various interactors [21–23]. Cell motility, in terms of spreading only, or even cell movement needs cross talk of various signaling events triggered by both cyto-, chemo- or hapto-taxis factors [24, 25], and other transmembrane (glyco)proteins (integrins and/or proteoglycans) [22, 24–26]. Moreover, cell motility, including cell prolongation extension (acting in cell spreading and locomotion), requires a high coordination of several events such as successive adhesions and detachments of the cells, by interacting with the extracellular matrix, and accompanied by changes in the cell shape eliciting cytoskeleton rearrangement [22, 27–30]. These events, that are highly controlled and modulated by the cell, result in an adequate mechanical behavior. All of the above mentioned events occur while the cells are involved in chemotaxis or haptotaxis. Cell motility was proven to depend on the strength of interactions between integrins and matrix proteins [28]. Both low adhesiveness and strong attachment to the matrix proteins impede the cell dynamics.

We report here results on the dynamics of TC versus fibroblasts on different matrix proteins, showing differences between the two types of cells in their ability to extend prolongations that depends on matrix proteins used to cover the culture surface.

## Materials and methods

### Cell culture

TC were investigated in a highly enriched culture of interstitial cells from mouse hearts. The hearts were dissected under a stereomicroscope, minced into millimetric pieces and dissociated by enzymatic digestion as described elsewhere [2]. Cultured TC in mouse heart were harvested using trypsin–EDTA in PBS and prepared for time-lapse videomicroscopy by suspending them at a density of 5 × 10^3^cells/ml in DMEM/F12 culture medium, supplemented with 10 % fetal calf serum and 100 U/ml penicillin—100 μg/ml streptomycin (all reagents from Sigma-Aldrich).

L929 mouse fibroblasts were grown in the same culture medium, but were harvested using PBS-EDTA and suspended for experiments at the same concentration, 5 × 10^3^cells/ml.

### Flow cytometry

**Fig. 1:**
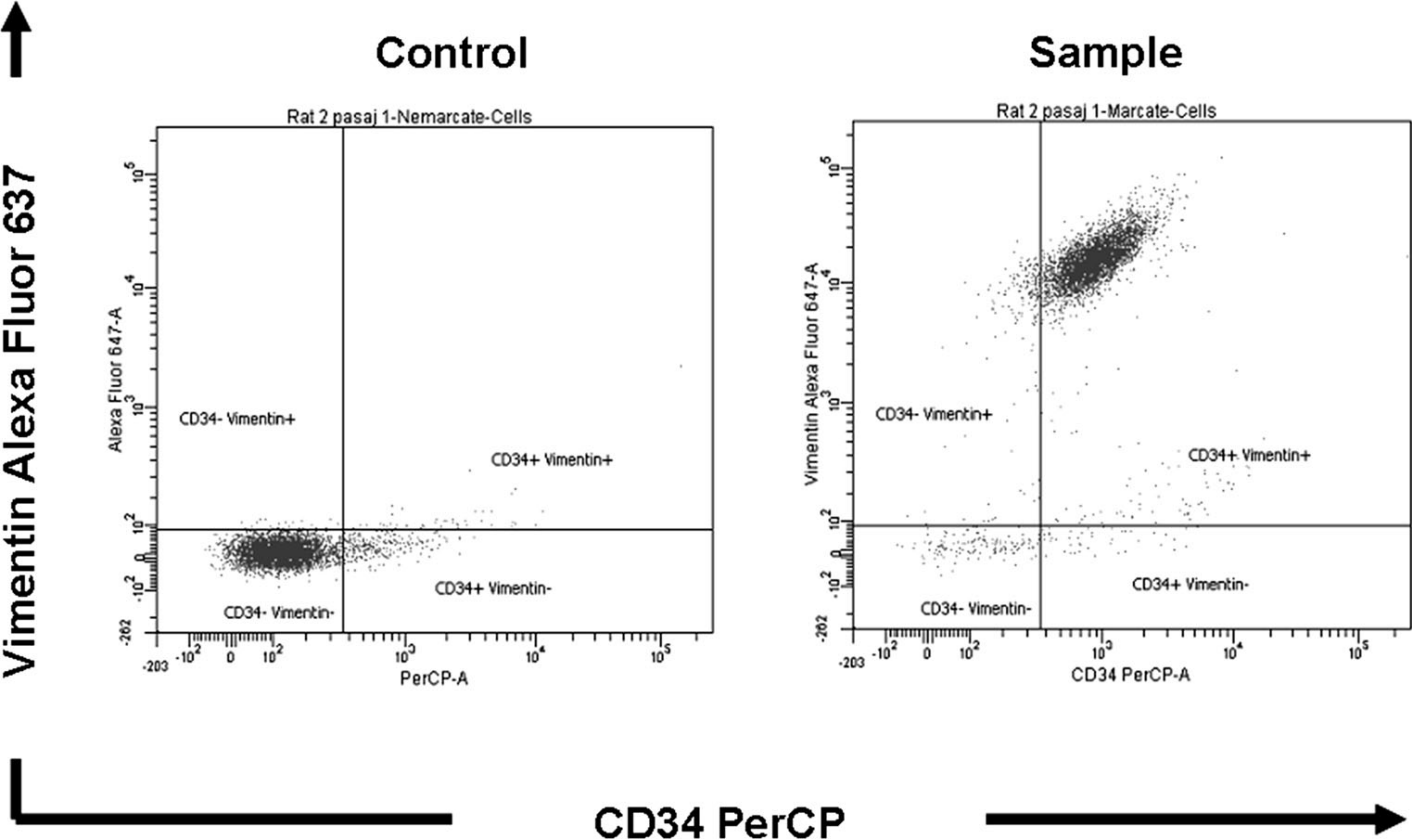
Representative results of flow cytometry assay by BD FACSCanto II cytometer for cultured interstitial cells obtained from rat heart. *Control* unlabeled cells. *Sample* cells labeled for rat vimentin and rat CD34

TC content in our enriched culture was assessed by double-labeling with Alexa Fluor 637-conjugated anti-vimentin monoclonal antibodies and PerCP-labeled anti-CD34 monoclonal antibodies (Santa Cruz Biotechnology Inc. Heidelberg, Germany) and analyzed in a BD FACSCanto II cytometer. Unlabeled cells were used as a control (Fig. 1) in order to avoid counting of false positive cells. Non-specific fluorescence signals due to spectral overlapping were automatically compensated for, using BD CompBeads particles. Data acquisition and analysis were performed with BD FACSDiva 6.1 software.

### Experiments for cell adherence and spreading by impedance measurements

Sixteen-well E-plates designed for the xCELLigence system (Roche Diagnostics, Mannheim, Germany) were prepared for adherence and spreading of cells in enriched interstitial cell samples and of fibroblasts as follows: four wells without any matrix protein (for control), four wells for collagen I (Coll), four wells for fibronectin (Fn), and four wells for laminin (Lam). Matrix proteins used for coating were solubilized in 0.1 M NaHCO_3_, at 5 μg/ml. Coating was done overnight, at 4 °C [31], by using 50 μl of matrix protein solution for each well. The wells were washed twice with plain medium, and background values of impedance were measured in serum-free medium, supplemented with 5 % bovine serum albumin (BSA-medium). Following this step, 2 × 10^3^ cells were added to each well, and the cell layer impedance was recorded for 18 h.

### Preparation of culture dishes

Culture dishes with four chambers (Hi–Q^4^ 35 mm Dishes, Ibidi GmbH, Martinsried, Germany) were used to simultaneously monitor the cell behavior in four different experimental conditions. These dishes were specially designed for BioStation IM (Nikon Corp. Kawasaki, Japan), equipment for time-lapse videomicroscopy experiments. The dishes were prepared as follows: (i) one chamber for control, without any matrix protein; (ii) the other three chambers were coated with Coll, Fn, and Lam, respectively. For coating, 300 μl of matrix protein solution at 5 μg/ml, in 0.1 M NaHCO_3_ were used overnight, at 4 °C. After washing with PBS, the dishes were ready for cell seeding in BSA-medium.

### Time-lapse videomicroscopy

Cells were seeded on the dish (300 μl cell suspension/chamber) and, after a 10 min incubation period at 37 °C and 5 % CO_2_, to promote cell adhesion, the dish was placed in BioStation IM to monitor adherence, spreading, and morphology dynamics of cells. Images were collected for 24 h every 5 min, in five different microscopic fields from each chamber. Data were collected from images by counting the number of moniliform prolongations, longer than the cell length, for cells present in the selected fields. To assess cell spreading the contours of cells were traced using the NIS Element BR software, which allows the user to determine the cell surface. Average values ± standard errors of media were plotted as a function of experimental time.

### Electron microscopy

Transmission electron microscopy (TEM) was performed on cultured cells (TC or fibroblasts) fixed with 2.5 % glutaraldehyde in 0.1 M cacodylate buffer, directly in the culture dish. The samples were post-fixed in 1 % OsO_4_ with 1.5 % K_4_Fe(CN)_6_ (potassium ferrocyanide-reduced osmium). Subsequently, cells were embedded in 1 % agar, dehydrated in graded ethanol series, and embedded in epoxy resin (Agar 100) [32]. The ultra-thin sections were cut with a diamond knife at a 60 nm thickness using an RMC ultramicrotome (Boeckeler Instruments Inc., Tucson, AZ, USA) and were double stained with uranyl acetate and lead citrate. Ultrastructural examination was performed with a Morgagni 286 transmission electron microscope (FEI Company, Eindhoven, The Netherlands) at 80 kV. Digital electron micrographs were recorded with a MegaView III CCD and iTEM-SIS software (Olympus, Soft Imaging System GmbH, Münster, Germany).

### Statistical analysis

Morphological data are presented as average values ± standard errors of media. Statistical significance was assessed by Student’s t test.

## Results and discussion

### Characterization of interstitial cells in mouse heart samples enriched in TC

**Fig. 2:**
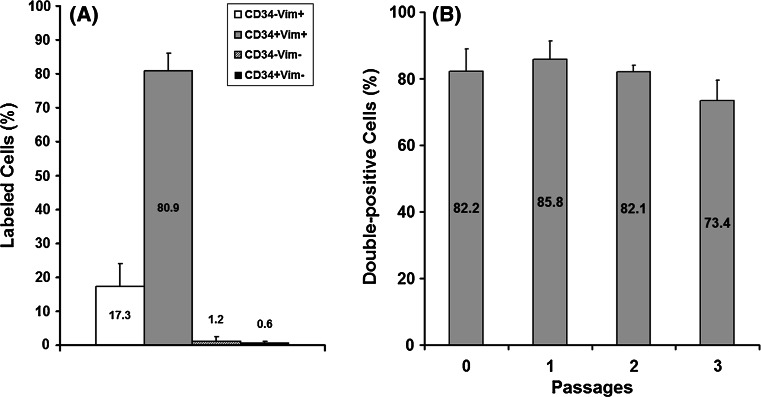
Results for flow cytometry analysis of rat heart interstitial cells; primary versus three successive passages in culture. **A** Average values as percent of cells labeled for CD34 and vimentin for four experiments at all passages. **B** Variation of percent of double-positive cells (CD34+vimentin+) along the four passages

Cells obtained from mouse hearts were tested by flow cytometry with double-labeling for vimentin and CD34 (Fig. 1), the markers proven to constantly characterize TC in many tissues [3, 15, 33]. We used flow cytometry for checking both homogeneity and phenotype of our isolated and cultured cells along the passages needed to our experiments to be done. Results showed that the cells are quite homogeneous in terms of morphological features (size and granularity), but by double-labeling for vimentin and CD34 the primary and cultured cell populations showed to be also highly homogeneous with only few percentages carrying a putative different phenotype. The results of four determinations during four cell passages revealed the presence in culture of double-positive cells (vimentin+CD34+) in an average proportion of 80.9 ± 5.3 % (Fig. 2A). Other cell types found in our samples were not double positive: CD34−vimentin+ (17.3 %), CD34−Vimentin− (1.2 %), and CD34+Vimentin− (0.6 %). The percent of double-positive cells slightly increased after the first passage in comparison with samples of primary cells, but the following three passages showed a slight downward trend: 82.2, 85.8, 82.1, 73.4 % (Fig. 2B). This decreasing trend for double-positive cells in culture along passages could be due to a putative low proliferation rate for TC. We did not chase the other cell types, such as CD34−vimentin+ (representing less than one out of five cells) in our time-lapse videomicroscopy experiments, to appreciate if they could be identified as different cells by morphological features. Moreover, nothing drew our attention in terms of different morphology of cells monitored in the experimental fields. This fact confirmed what flow cytometry showed regarding the homogeneity of major double-positive cell population (granularity and size).

In conclusion, according to our flow cytometry results, in the experiments reported in this paper TC were the main type of cells in culture.

### Dynamics of cell spreading

**Fig. 3:**
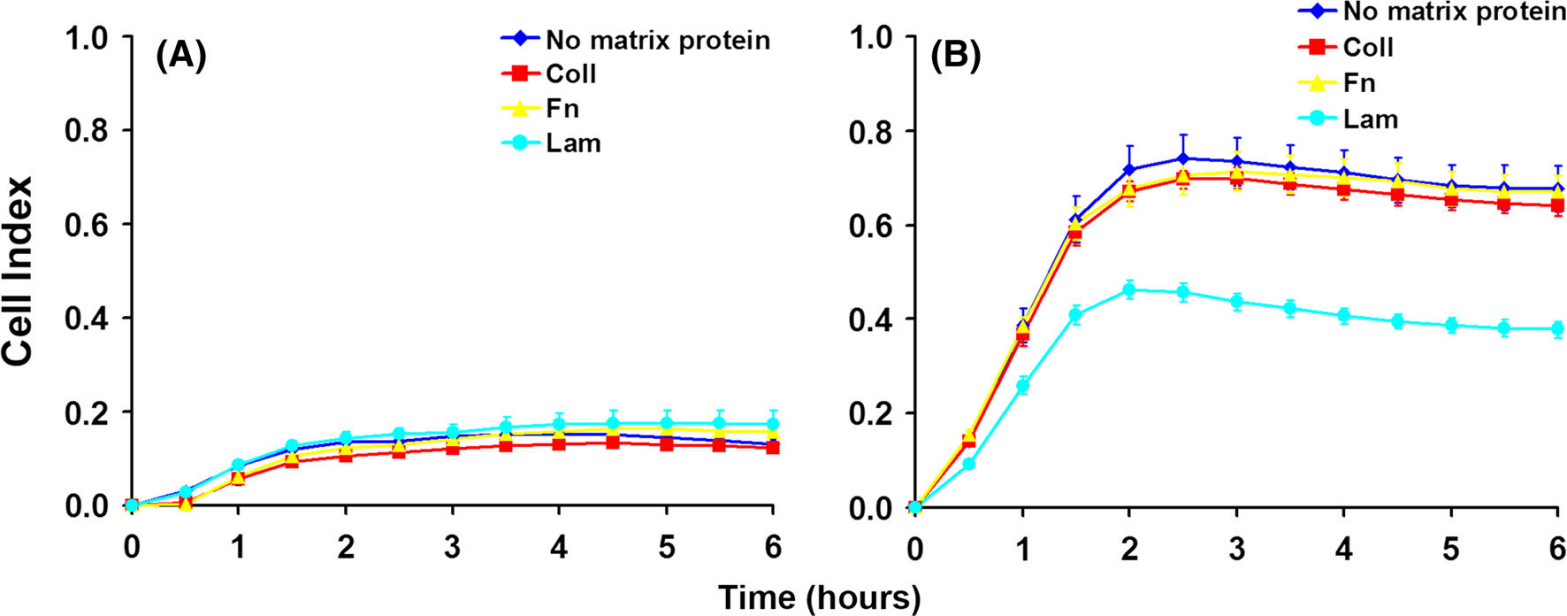
Dynamics of cell spreading on different matrix proteins, monitored by measurement of the cell layer impedance. **A** Spreading of L929 fibroblasts. **B** Spreading of telocytes

**Fig. 4:**
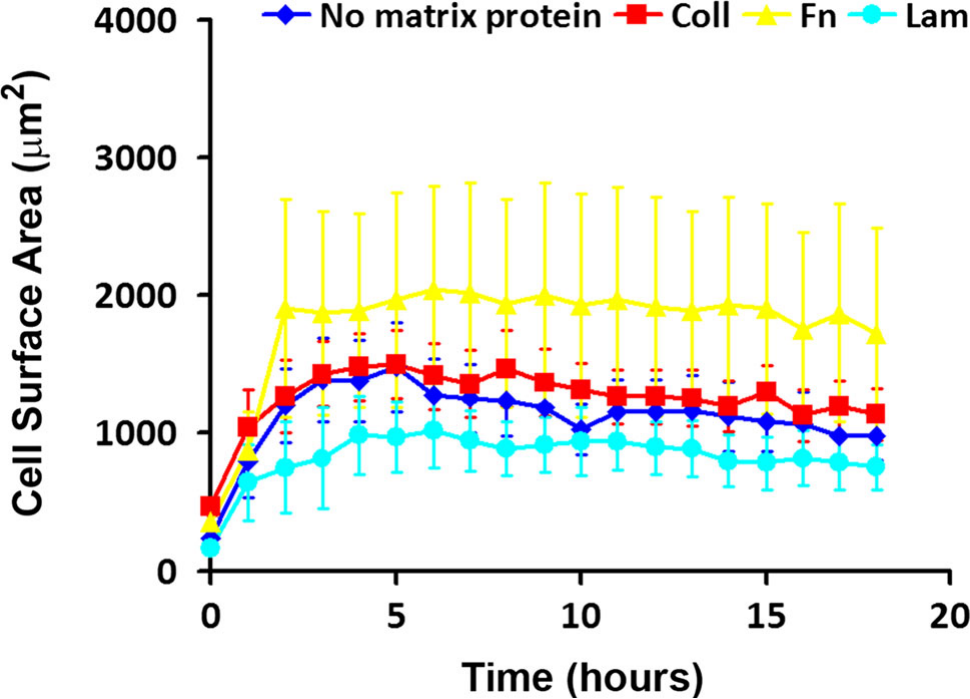
Dynamics of telocyte spreading on different matrix proteins evidenced by the measurement of the cell surface area in time-lapse videomicroscopy experiments. The variability of surface area for the same cell at different experimental time points explains the high error bars on the plots expressing the huge dynamics in cell morphology

For a cell to extend prolongations it needs to adhere and spread first. Cell adherence and spreading were monitored by measuring the impedance of the cell layer using the xCELLigence system. L929 fibroblasts showed a similar low spreading on every matrix protein and on the uncoated control surface (Fig. 3A). This fact suggests a rather non-specific adherence and spreading perhaps as a result of the production and secretion of the cells’ own matrix proteins, during the experiments. Cells in TC enriched samples proved a significantly lower adherence and spreading on laminin, while for control (no matrix), Coll, and Fn experiments, the adherence and spreading did not show differences (Fig. 3B). We reason that the observed differences could be explained as an effect of the diversity of cells in the samples. The global similar value of the impedance recorded when the cells were seeded on different matrix proteins could be due to the fact that the variations in the individual cell morphology dynamics are hidden by canceling each other. Therefore, we investigated the adherence and spreading of TC in time-lapse videomicroscopy experiments. The surface area of any identified TC was measured by tracing the contour of the cell shape, which resulted in a digital measurement done by the software application. According to this assessment, the results showed that TC have different spreading dynamics (Fig. 4).

TC spread in 2–4 h on every matrix protein with no significant difference in terms of spreading time. However, the spreading ability is different, depending on the matrix protein used for surface coating, by reaching different average spreading areas. The largest areas were obtained by spreading on Fn, while the smallest were noted on Lam. Spreading on Coll and in control experiments (no matrix protein on the culture surface) was quite similar. Fibroblasts showed a lower spreading dynamics needing 8–10 h in both control experiments, and on Coll, Fn, or Lam (not shown). Surprisingly, in our experimental conditions the spreading surface of the fibroblasts is growing continuously even at 18 h. Moreover, the fibroblasts seeded on a surface with no matrix and on Lam proved a lower ability to spread in terms of cell surface area, while the spreading on Coll and Fn seems to be faster and larger.

**Fig. 5:**
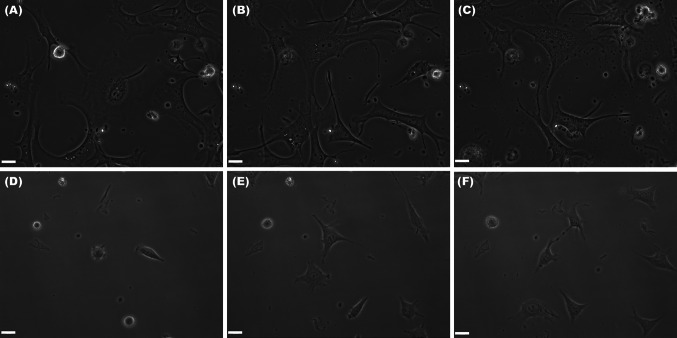
Spreading of telocytes (**A**, **B**, **C**) and fibroblasts (**D**, **E**, **F**) on collagen I. The size of telocytes is higher in comparison with that of fibroblasts at any experimental time point. **A** and **D** cell spreading at 4 h after seeding; **B** and **E** spreading at 12 h; **C** and **F** spreading at 18 h. *Bar* 20 μm

**Fig. 6:**
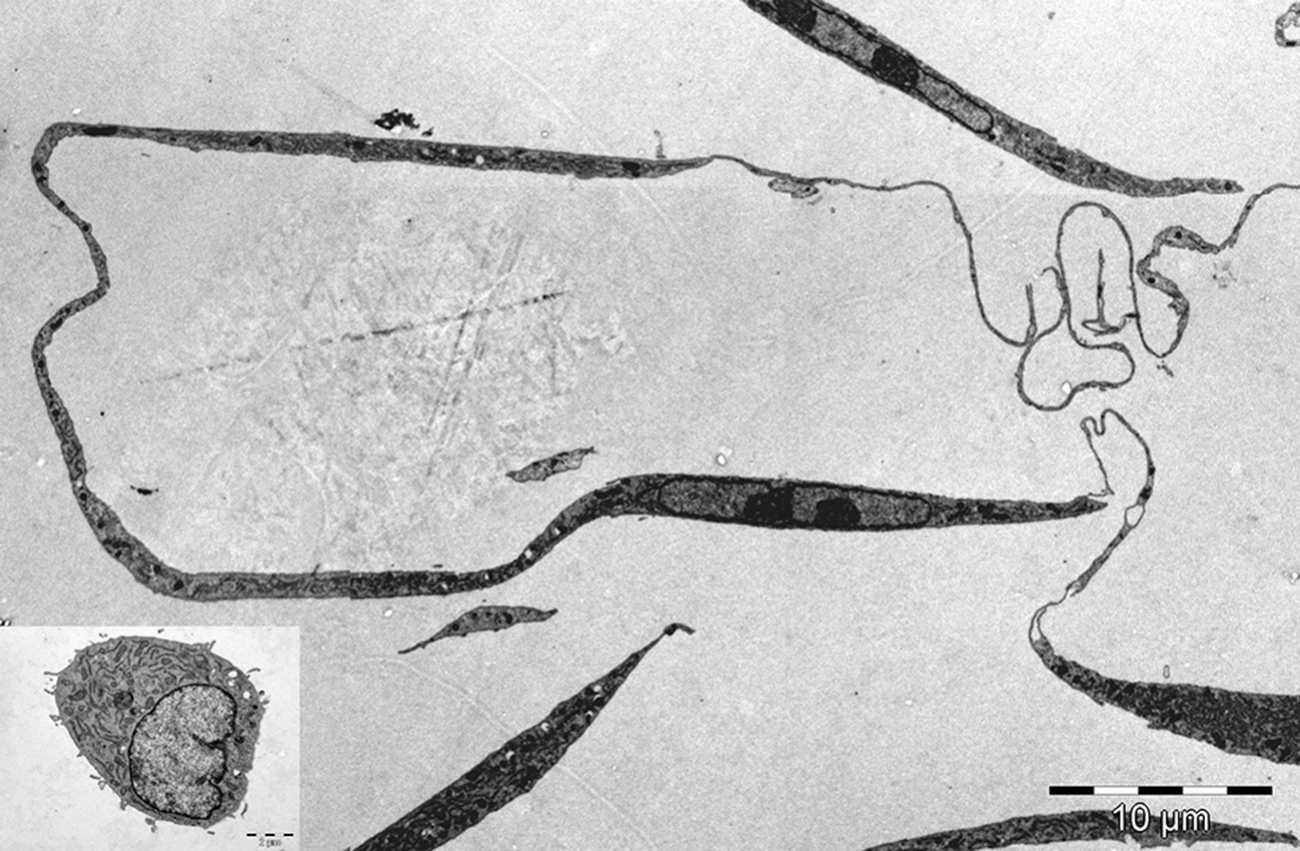
Electron micrographs comparing the morphological and ultrastructural features of telocytes and fibroblasts. The main image: a telocyte extending a convoluted telopode about 120 μm long, with podomers and podoms. *Inset* a fibroblast shown at the same magnification as the telocyte

The ability of TC to spread in any experimental conditions used was obviously higher (between ~800 and ~2,000 μm^2^), while fibroblasts spread on lower areas (200–400 μm^2^). The results are in accordance with the spreading ability registered in experiments monitoring the cell layer impedance. The dimensional differences between TC and fibroblasts are suggestively noted in phase microscopy images captured in BioStation IM (Fig. 5). The morphological differences observed for cells used in time-lapse videomicroscopy experiments have been also noted on cells prepared for TEM (Fig. 6). Moreover, electron micrographs’ analysis proved the utility of “platinum standard” [34] to differentiate between TC and fibroblasts, while the recent investigations reported differences for both gene profile and proteomics between cells in our culture, highly enriched interstitial cells from mouse heart and fibroblasts or other interstitial cells [16–18]. An up-to-date review is available [35].

### Dynamics of prolongations’ extension

**Fig. 7:**
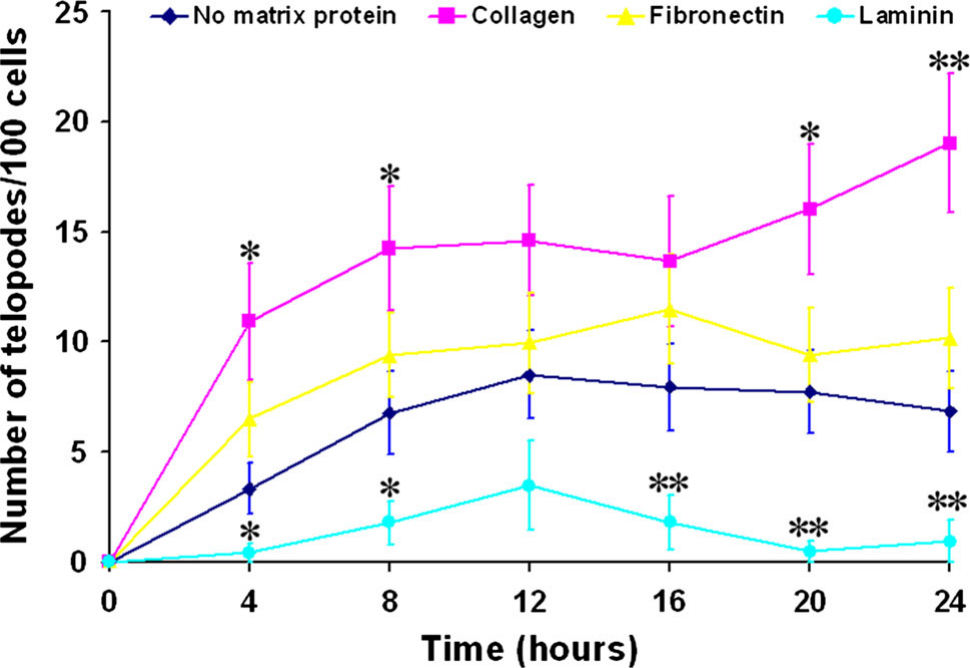
Dynamics of telocytes’ Tp extension on different matrix proteins used to prepare the surface culture compared to the standard culture surface (no matrix protein) as control. *Symbols* * (*p* < 0.05) and ** (*p* < 0.01) point out the statistical significance of the data at different experimental point times, in comparison with control values (no extracellular matrix protein covering the culture surface)

The highest ability of TC to extend Tp is proven when seeded on Coll, while the fewest number of prolongations appeared for cells on Lam (Fig. 7). That makes sense, because TC were never observed interacting with basal laminae, in any of the tissues investigated. The highest dynamics of telocytes’ Tp, on surfaces coated with collagen I, is supported by the lower adherence and spreading on this matrix protein, while the higher adherence and spreading on fibronectin impede TC to easily extend Tp. Therefore, the balance in the strength of forces developed by TC for adherence and spreading on collagen I allows the cells to extend Tp with a higher dynamics. We may hypothesize that collagen I, the most abundant matrix protein, present in most connective tissues and almost in all organs, could represent the best track for TC to direct the extending Tp toward the interaction partners, other cells in tissues, to organize, reorganize, or regenerate biological structures. Moreover, considering the observation in our experiments, cells do not usually extend simultaneously more than one Tp, we may affirm that the values in Fig. 7 represent percents of cells simultaneously extending Tp. Despite the fact that our samples are highly enriched in TC (around 80 % as proven by flow cytometry results, using double-labeling), only a fraction extends Tp at the same time, with the highest stimulation on collagen.

The mouse fibroblasts tested in our experiments showed a totally different morphology dynamics, in terms of extending cell processes. It is noteworthy, L929 fibroblasts showed a very low ability to extend even short and non-moniliform prolongations, on all tested matrix proteins. There was no prolongation longer then cell body noted. These results proved that TC in our samples of enriched interstitial cells from mouse heart and cultured fibroblasts behave differently.

## Conclusions

Our results showed TC seeded on various matrix proteins behave differently in terms of adherence, spreading, and dynamics of Tp extension. The highest telocyte adherence and spreading occurred on fibronectin, but the highest dynamics of Tp extension was noted for the cells seeded on collagen I. Comparatively, time-lapse investigation of TC and fibroblast behavior showed significant differences in terms of adherence, spreading, and ability to extend prolongations. This is the first comparative study about the dynamics of TC versus fibroblast morphology, confirming that our cultured cells obtained from mouse heart, as samples enriched in interstitial cells, contain cells that behave differently as compared with the mouse fibroblast cell line L929.
